# New insights into the role of Klotho in inflammation and fibrosis: molecular and cellular mechanisms

**DOI:** 10.3389/fimmu.2024.1454142

**Published:** 2024-09-06

**Authors:** Xinyue Zhao, Donghe Han, Chun Zhao, Fengfan Yang, Zhimei Wang, Yujiao Gao, Meihua Jin, Ran Tao

**Affiliations:** ^1^ Chronic Disease Research Center, Medical College, Dalian University, Dalian, Liaoning, China; ^2^ Department of Anatomy, Medical College, Dalian University, Dalian, Liaoning, China; ^3^ Department of Immunology, Medical College, Dalian University, Dalian, Liaoning, China

**Keywords:** anti-aging, inflammation, tissue fibrosis, Klotho, therapy

## Abstract

As the body’s defense mechanism against damage and infection, the inflammatory response is a pathological process that involves a range of inflammatory cells and cytokines. A healthy inflammatory response helps the body repair by eliminating dangerous irritants. However, tissue fibrosis can result from an overly intense or protracted inflammatory response. The anti-aging gene Klotho suppresses oxidation, delays aging, and fosters development of various organs. Numerous investigations conducted in the last few years have discovered that Klotho expression is changed in a variety of clinical diseases and is strongly linked to the course and outcome of a disease. Klotho functions as a co-receptor for FGF and as a humoral factor that mediates intracellular signaling pathways such as transforming growth factor β (TGF-β), toll-like receptors (TLRs), nuclear factor-kappaB (NF-κB), renin -angiotensin system (RAS), and mitogen-activated protein kinase (MAPK). It also interferes with the phenotype and function of inflammatory cells, such as monocytes, macrophages, T cells, and B cells. Additionally, it regulates the production of inflammatory factors. This article aims to examine Klotho’s scientific advances in terms of tissue fibrosis and the inflammatory response in order to provide novel therapy concepts for fibrotic and inflammatory disorders.

## 1 Introduction

**Figure 1:**
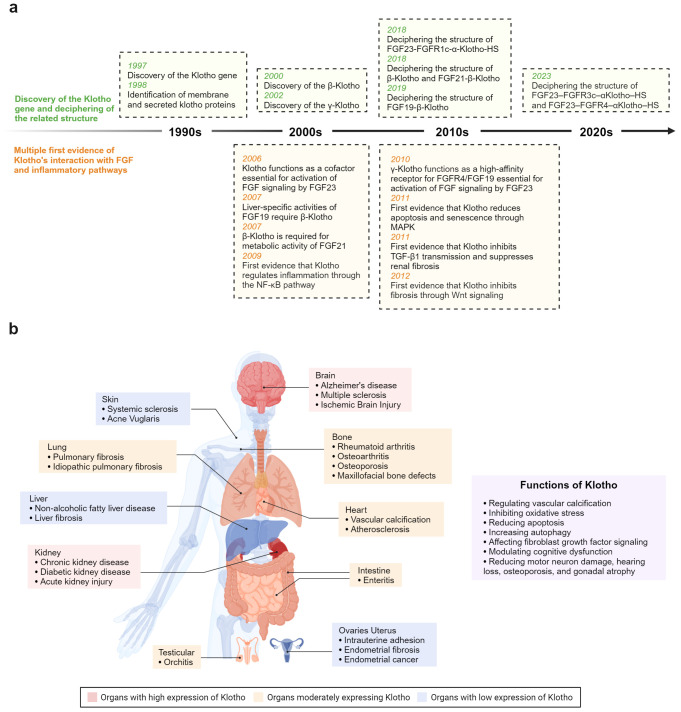
**(A)** Timeline depicting key breakthroughs in the field of anti-aging protein Klotho. **(B)** Klotho plays a crucial role in modulating inflammation and fibrosis across various organ systems.

An inflammatory reaction is triggered by the stimulation of infection or endogenous signals associated with the abnormality of structure, function, or metabolism in various tissues (1). There are known to be two types of inflammatory responses. A healthy inflammatory response helps the body repair by eliminating dangerous factors. If the body’s self-regulation ability is disturbed, permanent chronic inflammation and tissue damage cause excessive activation (or polarization) of (muscle) fibroblasts and excessive deposition of extracellular matrix, causing fibrosis of various tissues and organs and leading to structural destruction and functional abnormalities of organs, until organ failure and poor prognosis (2–4). In addition to disturbances in immune homeostasis caused by autoimmunity and infection, the aging process is frequently linked with systemic inflammatory syndromes, also known as inflammatory aging. Comparative genetic analysis of tissues from young and aged mice, rats, and humans has revealed that genes related to inflammation and immune response play a significant role in driving age-associated changes in gene expression (5). The modulation of the equilibrium between pro- and anti-inflammatory signaling by anti-aging genes can potentially prevent or delay the onset of inflammatory and fibrotic diseases. Klotho (6), a key regulatory protein in aging-related disorders, exhibits high expression levels in various organs such as the kidney (7), brain (8), reproductive organs (9–11), pituitary gland (12), and parathyroid glands (13). A more profound understanding of Klotho’s biology has unveiled its multifaceted functions that extend beyond mere “anti-aging.” Studies have demonstrated that Klotho serves as a “beneficial” factor in various organs, including the kidney (14, 15), heart (16, 17), lungs (18, 19), blood vessels (20, 21), liver (22, 23), brain, and reproductive organs. It regulates calcium and phosphorus metabolism, impacts vascular calcification, and contributes to a range of biological processes such as inflammation, oxidative stress, and apoptosis (**Figure 1**). In this comprehensive review, our objective is to furnish a thorough overview of the structural and functional characteristics of the anti-aging gene Klotho. We aim to delve into its impact on the phenotype and function of inflammatory cells such as macrophages, T cells, B cells, etc., as well as its intricate interactions with FGF and intracellular signaling pathways (TGF-β, TLRs, NF-κB, RAS, and MAPK, etc.) in inflammatory and fibrotic disease. Furthermore, we will summarize current methods for reinstating Klotho levels and activity. Lastly, we will examine Klotho assays and conclude with an analysis of the potential application of Klotho as a biomarker for the diagnosis and treatment of inflammatory and fibrotic diseases.

## 2 Klotho structure

**Figure 2:**
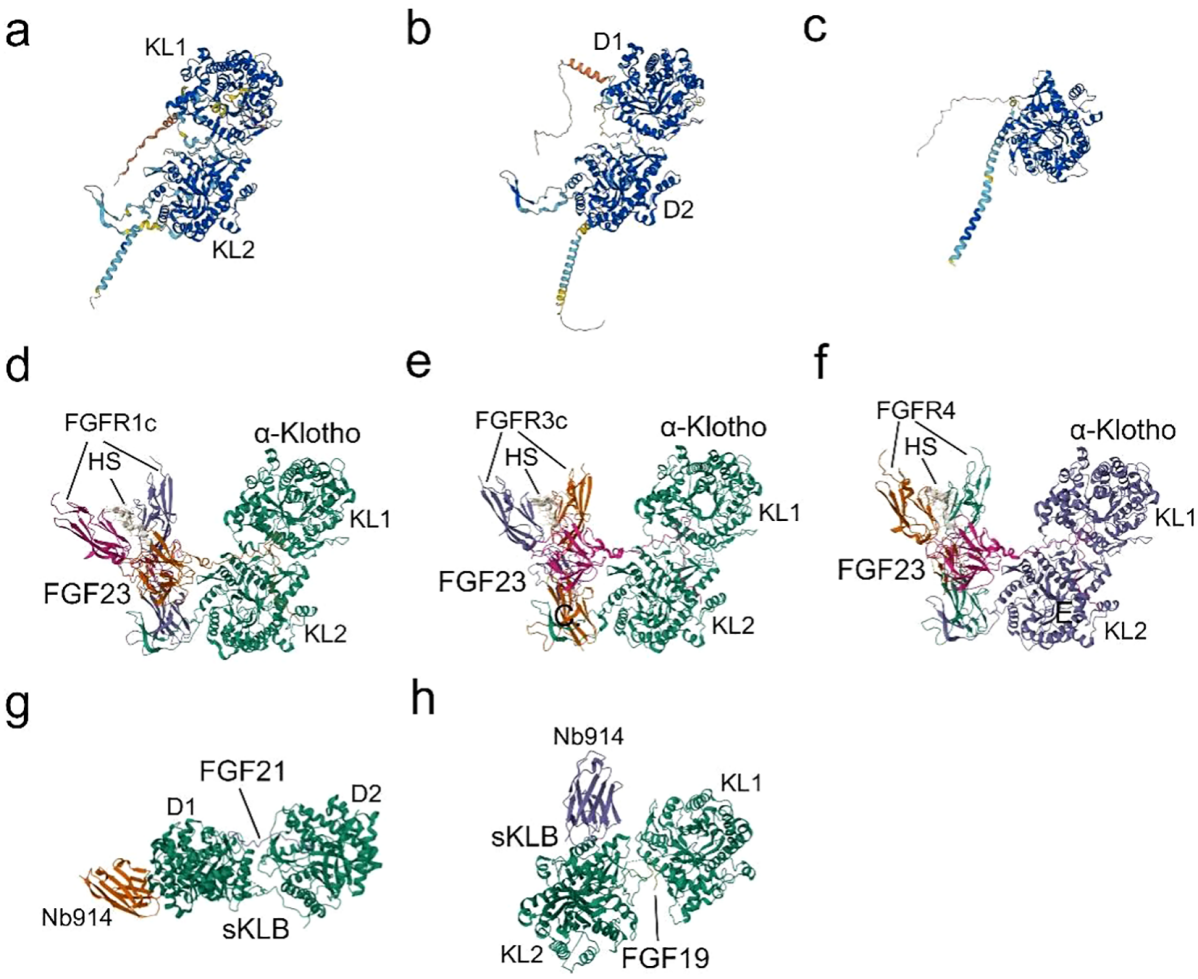
Structures and Binding of Klotho to FGFs: **(A)** The predicted structure of α-Klotho, as determined by AlphaFold (AF-Q9UEF7-F1) (32, 33). **(B)** The predicted structure of β-Klotho, as determined by AlphaFold (AF-Q86Z14-F1) (32, 33). **(C)** The predicted structure of γ-Klotho, as determined by AlphaFold (AF-Q6UWM7-F1) (32, 33). **(D)** Cryo-EM structure of the Quaternary complex FGF23-FGFR1c-aKlotho-HS, derived from PDB accession code 7YSH (34). **(E)** Cryo-EM structure of the Quaternary complex FGF23-FGFR3c-aKlotho-HS, derived from PDB accession code 7YSU (34). **(F)** Cryo-EM Structure of the Quaternary complex FGF23-FGFR4-aKlotho-HS, derived from PDB accession code 7YSW (34). **(G)** X-ray diffraction crystal structure of β-Klotho bound with FGF21, derived from PDB accession code 5VAQ (35). **(H)** X-ray diffraction crystallography depicting the complexes formed between Beta-Klotho and the C-terminal peptide of FGF19, obtained from PDB accession code 6NFJ (36).

The Klotho gene is located in the 13q12 region (humans and mice) or 12q12 region (rats) and comprises four introns and five exons encoding three subtypes—Klotho: α-, β-, and γ-Klotho (**Table 1**) (24). α-Klotho is highly expressed in the kidney, choroid plexus, parathyroid glands, and sinus node, as well as in the nervous, respiratory, digestive, and reproductive systems to varying degrees (25). It comprises two extracellular domains (KL1 and KL2), a transmembrane segment (TM), and a short non-signaling cytoplasmic tail (CYT) (**Figure 2**). α-Klotho is divided into α-Klotho™ and α-Klotho^ecto^ (26). α-Klotho™ forms a ternary receptor complex with fibroblast growth factor receptor 1c (FGFR1c), FGFR3c, FGFR4, and FGF23 (**Figure 2**), mediating the balance of calcium and phosphorus metabolism and active vitamin D production in the body (26). Selective shearing (both α and β shearing modes) of α-Klotho™ in the kidney’s distal tubules is caused by a disintegrin and metalloproteinase 10 (ADAM10), ADAM17, and the β-site amyloid precursor protein cleaving enzyme 1 (BACE1), which yields α-Klotho^ecto^ (27). With two tandem glycoside hydrolase-like (GH) structural domains, D1 and D2 (**Figure 2**), β-Klotho is mostly expressed in the liver and white adipose tissues (pancreas, brain), where it can form complexes with FGFR1c and FGFR4 and trigger downstream signaling pathways (28, 29). γ-Klotho has only one extracellular structural domain similar to β-glucosidase and a short cytoplasmic tail region, and is mainly expressed in kidney, brown fat, skin, and eyes (30), and becomes a high-affinity receptor for FGFR4/FGF19 after binding to FGFR1b, FGFR1c, and FGFR2c (31). Since the research on γ-Klotho is still in the initial stages and the biological role of γ-Klotho is not clear, this paper mainly reviews the role of the other two subtypes, α-Klotho versus β-Klotho.

**Table 1:** Three different kinds of Klotho.

Types	Receptors	Areas	Structures	Distributions	References
α-Klotho	FGF23-FGFR1c/ FGFR3c/ FGFR4(mKL); FGF23-FGFR1c(sKL)	12q12(Rat) 13q12(Human and mice)	Two extracellular domains (KL1 and KL2), a transmembrane segment (TM), and a short non-signaling cytoplasmic tail (CYT)	Kidney, parathyroid glands, sinus node, nervous, respiratory, digestive, and reproductive systems	(6, 26, 27)
β-Klotho	FGF21-FGFR1c; FGF15/19-FGFR4	4q	Two tandem glycoside hydrolase-like (GH) structural domains, D1 and D2	Liver and white adipose tissue (pancreas, brain)	(28, 29)
γ-Klotho	FGF19-FGFR1b/ FGFR1c/FGFR2c/ FGFR4	Unknown	One extracellular structural domain similar to β-glucosidase and a short cytoplasmic tail region	Kidney, brown fat, skin, and eyes	(30, 31)

## 3 Klotho regulates fibrosis and inflammation through inflammatory cells

Immune cells are vulnerable to endogenous DNA damage during the process of aging, and the senescent cells eventually lose their capacity to eradicate pathogens and damaged cells. Meanwhile, the accumulation of senescent cells usually downregulates the expression of proliferation-related proteins and increases a large number of inflammatory factors; then, the status is defined as low-grade chronic inflammation. Persistent low-grade chronic inflammation could lead to fibrosis of injured tissues and result in the dysfunction of organs. Therefore, modulating inflammation by regulating senescent immune cells with anti-aging medications, such as Klotho, might ameliorate fibrotic diseases (**Table 2**) (37).

**Table 2:** Klotho regulates inflammatory cells.

Cells	Cytokines	Effects	Places	References
Macrophage	↓:CD206, IL-10, TGF-β	Inhibits the TLR4/MyD88/NF-κB pathway; Induces RAW264.7 polarization to M2a/M2c; Exerts anti-inflammatory effects and protects the heart	Heart	(43)
	↓:F4/80 and CD68 co-tagged M1;MCP-1 and ICAM-1	M1 decrease; Alleviating aortic valve fibrosis and vascular inflammation	Aorta, blood vessels	(44)
	↓:IL-1β, TNF-α, IL-6	Promoting macrophage M2 polarization; Increasing the expression of anti-inflammatory factors; Alleviates renal fibrosis and cardiac hypertrophy	Kidney, heart	(45)
	↓:iNOS, MDA ↑:SOD enzyme	Increased M2a/M2c Significantly suppressed inflammatory response and oxidative stress levels	Kidney, brain	(46)
	↓:IL-6 and IL-1β ↑:IL-10	Inhibits M1 polarization Promotes M2 polarization through paracrine secretion; Exerts anti-inflammatory effects	Bone	(47)
T cell	↓:IL-17, IL-6, IL-23, IFN-γ	Inhibits Th1 and Th17 cell differentiation; Promotion of (at least some) Treg cell differentiation; anti-inflammatory	Brain	
	↓:IGF-1R, AKT, ERK1/2	Regulation of T-cell lymphoma proliferation, anti-cance	Lymph	(53)
B cell	↓:VCAM1, PCAM, serum CRP, TNF-α, IL-1β, TGF-β1, collagen-1, sclerenchyma, MMP-2, MMP-9	Restoration of B-cell populations and serum IgG levels to alleviate vascular inflammation and arterial remodeling	Vessel, artery	(56)
Endothelial cell	↓:ROS, TNF-α, IL-6	Mitigation of high glucose-induced glomerular endothelial cell injury	Kidney	(57)

### 3.1 Monocyte-macrophage

Macrophages originate from hematopoietic stem cells in the bone marrow and undergo differentiation from circulating monocytes in the peripheral circulation (38). Upon stimulation by various factors, such as the microenvironment, macrophages can polarize into distinct phenotypes (primarily M1 and M2) (39, 40), eliciting a Th1-type immune response (41) or promoting a Th2-type immune response (42).

This results in their display of both pro- and anti-inflammatory activities. Recently, studies have demonstrated that Klotho could regulate the polarization of macrophages. For example, Klotho could show anti-inflammatory effects by blocking the Toll-like receptor 4 (TLR4)/myeloid differentiation factor 88 (MyD88)/nuclear factor-kappaB (NF-κB) pathway—which causes a large increase in the production of CD206, IL-10, TGF-β, and polarizes RAW264.7 to M2a/M2c, eventually ameliorating cardiac aging (43). Following the restoration of secreted Klotho (sKL) expression, studies found a decrease in F4/80 and CD68 co-tagged M1, a suppression of monocyte chemoattractant protein-1 (MCP-1) and intercellular adhesion molecule 1 (ICAM-1) expression on the aortic valves of senescence-accelerated mice prone strain 1 (SAMP1) mice, and a reduction in aortic valve fibrosis along with aging-associated vascular inflammation (44). Furthermore, by enhancing M2 polarization and upregulating the expression of anti-inflammatory proteins, Klotho overexpression in macrophages attenuates fibrosis and cardiac hypertrophy and decreases the expression of pro-inflammatory factors (IL-1β, TNF-α, and IL-6) (45). In the kidneys and brains of elderly mice, researchers discovered that the peripheral delivery of Klotho enhanced M2a/M2c, lowered malondialdehyde (MDA) and iNOS levels, enhanced superoxide dismutase (SOD) enzyme activity, and markedly reduced oxidative stress and inflammatory responses (46). Through interactions between stem cells and macrophages, Klotho can affect macrophage polarization in addition to directly affecting the macrophages. According to Niu et al. (47), pretreating human periodontal stem cells (hPDLSCs) with Klotho had anti-inflammatory effects by promoting macrophage M2 polarization through paracrine secretion and inhibiting macrophage M1 polarization. Thus, by affecting the direction of macrophage polarization and inhibiting the production of inflammatory factors, Klotho exerts an antagonistic effect on systemic inflammation and alleviates fibrosis at multiple sites, including the heart, kidney, and brain.

### 3.2 T cells

T lymphocytes are the main effector cells in cellular immunity. They produce cytokines during the immune response, which can influence other immune cell types and mediate inflammatory reactions (48, 49). Based on cell surface markers, T cells are divided into two subpopulations: CD4^+^ and CD8^+^. The CD4^+^/CD8^+^ ratio reflects immune activation and chronic inflammation as drivers of disease (50).

Klotho levels have been shown to serve as an independent predictor of the CD4^+^/CD8^+^ ratio (51). Low Klotho levels enhance MDA and buildup oxidative stress, which in turn increases CD4^+^ T cell counts (51). Large T-cell infiltration was observed in KL(+/-) mice (52), and it was discovered that Klotho can contribute to fibrosis and inflammation by influencing T-cell differentiation. Our earlier research revealed that Klotho may suppress the inflammatory response and alter the pathological process as multiple sclerosis develops. This is achieved by suppressing Th1 and Th17 cell differentiation, encouraging the differentiation of a few Treg cells, and lowering the secretion of the main pro-inflammatory cytokines, IL-17, IL-6, IL-23, and IFN-γ. As an endogenous circulating hormone that effectively inhibits tumor growth, Klotho can cause carcinogenesis when it persists or is present in excess. Patients with T-cell lymphoma have much lower levels of Klotho in their lymph nodes than in normal lymph nodes; the overexpression of Klotho can control the growth of T-cell lymphomas and decrease insulin-like growth factor-1 receptor (IGF-1R) signaling, which has anticancer properties (53).

### 3.3 B cells

B lymphocytes are pluripotent stem cells derived from bone marrow that, in addition to producing antibodies, secrete inflammatory mediators to drive the inflammatory response (TNF-α, IL-6, etc.) and inhibit hyperinflammation by metabolizing extracellular adenosine triphosphate (ATP) to adenosine diphosphate (ADP) and secreting IL-10 (54).

In comparison to wild-type mice, KL (-/-) animals exhibited thymic atrophy, severe B-lymphocytopenia, and considerably lower B-cell counts in bone marrow and peripheral blood (55). It has been discovered that Klotho may regulate the synthesis of negative regulatory factors, the lack of which modifies positive signaling for B lymphopoiesis, modifies the hematopoietic microenvironment’s three-dimensional structure or inhibits specific cell types crucial for B lymphopoiesis, and suppresses the bone marrow’s ability to produce B lymphocytes (55). According to Fan et al. (56), AAV-based delivery of sKL gene (AAV-sKL) effectively alleviated aging-related vascular inflammation and arterial remodeling in the aorta of aged mice by reducing SAMP1 and inflammatory cell infiltration, thereby inhibiting the expression of TGF-β1, collagen-1, sclerenchyma, MMP-2, and MMP-9, and partially by restoring B-cell populations and serum IgG levels.

### 3.4 Other cell types

Apart from the inflammatory cells already mentioned, Klotho can affect fibroblasts, endothelial cells, and other cells. Klotho protects the periodontium in a high-sugar environment, increases the capacity of periodontal fibroblasts to scavenge reactive oxygen species (ROS), and has anti-apoptotic effects on periodontal fibroblasts. The overexpression of Klotho in diabetic nephropathy (DN) reduces the damage caused by excessive glucose in human glomerular endothelial cells (HRGECs) (57). Moreover, Klotho has anti-apoptotic and anti-aging properties when it activates superoxide dismutase, which shields endothelial cells from the damaging effects of oxidative stress. Pericytes control the release of neutrophils and interact with endothelial cells to influence the permeability of blood vessels. In addition to causing pathological alterations, such as pericyte loss and degeneration, inflammatory stimuli also lead pericytes to express pertinent components that worsen inflammation. However, more research is required to fully understand the mechanism underlying the roles of pericytes and Klotho in fibrosis and the inflammatory response. When combined, Klotho can alter the phenotypic and function of inflammatory cells, thereby regulating fibrosis and the inflammatory response of the body.

## 4 Klotho utilizes inflammatory pathways to control fibrosis and the inflammatory response

### 4.1 FGF pathway

FGFs are pleiotropic molecules that further trigger a variety of cellular processes, including inflammation, angiogenesis, cell proliferation, apoptosis, metastasis, and wound repair by combining with four tyrosine kinase FGFRs (58). Since Kurosu et al. (59) found the structure of Klotho as being a cofactor of FGF, an increasing number of reports have demonstrated the functions of Klotho in cellular processes by forming complexes with different FGF/FGFR.

α-Klotho first binds to the hydrophobic pocket in the D3 region of FGFRs (FGFR1c-3c and FGFR4) through the β1-α1 loop, forming a receptor binding arm (RBA) (26), while a large cleft at the junction between KL1 and KL2 embraces FGF23’s long C-tail (**Figure 2**). β-Klotho-FGFR1 interacts with FGF21 N-terminus through the D2-D3 region, and the D1 region exerts a β-Klotho-FGFR1 complex as well as a FGF21-FGFR1-β-Klotho complex formation negative regulatory role (35, 60). Due to the substitution of a crucial glutamate in the molecule, KL1 and KL2 of the extracellular ligand-binding region of β-Klotho cannot perform glycoside hydrolase activity. Instead, KL1 and KL2 engage with two highly conserved areas in the C-terminus of FGF19 and FGF21 (**Figure 2**). Since the C-terminal structures of FGF19 and FGF21 are 40% identical and share binding sites in the β-Klotho structure, there is no synergistic interaction between the two proteins (61). As an extra co-receptor, HS causes FGFR dimerization and activation upon the establishment of a stable FGF-FGFR-Klotho ternary complex (**Figure 2**). This initiates significant metabolic activities of the FGFR (26), which are linked to the processes of growth and development, wound healing, fibrosis, and inflammatory production.

#### 4.1.1 α-Klotho and FGF23

Changes in FGF23 and Klotho protein levels play a role in promoting fibrosis through various mechanisms, including oxidative stress, inflammatory responses, and activation of the renin-angiotensin system (RAS). These changes can occur directly or indirectly and lead to structural and functional alterations in organs. In response to inflammatory stimuli, Rodríguez-Ortiz et al. (61) observed that chronic kidney disease (CKD) mice exhibited increased FGF23 expression and lower levels of circulating Klotho protein. Additionally, *in vitro* tests have revealed that inflammatory circumstances decrease Klotho levels while stimulating FGF23 expression via the activation of NF-κB and Wingless/integrated (Wnt)/β-catenin signalings (61). By suppressing Wnt signaling, sKL can reduce collagen deposition and the generation of reactive oxygen species, reduce cardiac fibrosis, and prevent the elevated expression of β-catenin and TGF-β brought on by FGF23 stimulation (62). Moreover, the type 2 ryanodine receptor (RyR2) hyperphosphorylation and arrhythmias are caused by FGF23-mediated stimulation of intracellular Ca^2+^ and Ca-calmodulin-dependent kinase II (CaMKII) (63). Combining α-Klotho and FGF23 can further reduce the harmful effects of FGF23 on the heart by inhibiting CaMKII, reducing arrhythmias, and activating the MAPK signaling pathway (64, 65).

#### 4.1.2 β-Klotho and FGF15/19

FGF19 is an intestinal factor that is induced by the bile acid-activated farnesol X receptor (FXR) and subsequently forms the FGF19-FGFR4-β-Klotho complex in the liver via the portal vein (66, 67). This complex regulates extracellular signal-related kinases 1 and 2 (ERK1/2) and other downstream kinases and lowers the synthesis of cholesterol 7a-hydroxylase (CYP7A1), the rate-limiting enzyme for bile acid synthesis, which in turn lowers bile acid production and prevents the emptying of the gallbladder. Increased IL-1β through c-Jun N-terminal kinase (JNK) and NF-κB pathways can suppress the expression of β-Klotho in the liver, thereby altering bile acid metabolism and resulting in problems of the intestinal-liver axis. In addition, infections of the intestines and liver cause an excessive release of inflammatory substances (67). In mice lacking β-Klotho, the FGF15-FGFR4-β-Klotho pathway is impeded, thereby resulting in elevated bile acid synthesis and secretion. This leads to modifications in the composition of bile acid, bile acid transporter proteins, and intestinal microbiota (reduced thickwell proportion and increased aspergillus proportion) (68–70). These alterations cause inflammation and fibrosis in the liver, subsequently impacting the kidneys, intestine, etc. (68). Pediatric non-alcoholic fatty liver disease (NAFLD) patients experience hepatic injury and an increased risk of ballooning and lobular inflammation as a result of the organism downregulating β-Klotho through the expression of the rs17618244 G>A β-Klotho variant in human liver carcinoma (HepG2) and human HCC cell line (Huh7 cells). In turn, this causes intracellular lipid accumulation and upregulates the expression of pro-inflammatory genes, including p62, acyl-CoA oxidase 1 (ACOX1), acyl-CoA synthetase long-chain family member 1 (ACSL1), IL-1β, and TNF-α. According to additional research, people with β-Klotho mutations have lower levels of FGF19 than those without the mutation (69). Consequently, β-Klotho protects hepatocytes against lipotoxicity and inflammation, and the FGF19-FGFR4-β-Klotho pathway is important in the pathophysiology of NAFLD (71).

#### 4.1.3 β-Klotho and FGF21

FGF21, a stress-inducible hormone predominantly produced in the liver and adipose tissue, is well-known for its role as a pleiotropic regulator of glucolipid metabolism and insulin sensitivity (72). Recent evidence suggests that plasma FGF21 levels are significantly correlated with fibrosis progression (73), and targeting the FGF21/FGFR/β-Klotho pathway has the potential to arrest or reverse inflammation and fibrosis in tissues such as the heart and skin (74). The reduced expression of FGF21, a cardiac kinin released under pathological stress, along with its co-receptor β-Klotho, can lead to FGF21 resistance and exacerbate the compensatory response to cardiovascular disease (75). In the diabetic heart, FGF21-mediated activation of cardiac FGFR1-β-Klotho induces cardiomyocyte secretion of MCP-1/CCL2 and promotes the expression of Vcan, inhibiting M1 polarization in infiltrating monocytes while mitigating inflammation in the diabetic heart (76). As a peptide hormone that enhances mitochondrial function and energy metabolism, the absence of β-klotho leads to reduced sensitivity to FGF21. Delivery of β-Klotho to the heart using ultrasound-targeted microbubble destruction (UTMD) technology enhances sensitivity to FGF21 by up-regulating Nrf2 target proteins such as heme oxygenase 1 (HO-1), NAD(P) H quinone dehydrogenase 1 (NQO1), glutamate-cysteine ligase modifier subunit (GCLM), effectively alleviating post-infarction-induced myocardial oxidative stress and mitochondrial injury, thereby significantly ameliorating cardiac insufficiency’ adverse remodeling (77). In terms of skin inflammation, the FGF21-FGFR1-β-Klotho receptor complex in the dermis significantly reduced the expression of pro-inflammatory factors (IL-1β, IL-6, IL-8, and TNF-α) in human immortalized keratinocytes (HaCAT) and inhibited inflammation induced by Cutibacterium acnes (C. acnes) (78). However, the antifibrotic mechanism of FGF21/FGFR/β-Klotho in other organs such as the liver, kidney, and lung remains to be discovered.

Klotho significantly enhances the binding of endocrine fibroblast growth factor (eFGF) to FGFR and synergistically boosts the activity of the eFGF signaling pathway. However, there are differences in the roles of Klotho and paracrine FGFs. Specifically, FGF2 exerts significant pro-proliferative and differentiation effects and plays a crucial role in promoting epithelial-mesenchymal transition (EMT) in renal tubular epithelial cells (79). Guan X et al. (80) discovered that Klotho inhibits the activity of the FGF2 signaling pathway by competitively binding to FGFR1. This inhibition leads to a reduction in FGF2-induced phosphorylation of fibroblast growth factor receptor substrate 2α (FRS2α) and activation of ERK1/2, as well as preservation of E-cadherin in renal tubules. Furthermore, Klotho inhibits the expression of fibronectin (FN) in the interstitium of obstructed kidneys, thereby suppressing the proliferation of interstitial fibroblasts and attenuating kidney fibrosis.

In conclusion, there has been a growing body of research focusing on the interaction between Klotho and eFGFs. However, further comprehensive exploration is needed regarding the regulatory mechanism of Klotho with paracrine FGFs. Investigating the composition and binding mechanisms of FGFs, Klotho, and FGFRs can potentially lead to the development of more effective antagonists and agonists (81, 82). To effectively prevent and treat fibrotic diseases, numerous clinical studies are required to understand the crosstalk effects of Klotho in various tissues and organs, as well as to further validate the safety and efficacy of targeting the Klotho-FGFs axis therapeutically.

### 4.2 Inhibition of the TGF-β signaling pathway

TGF-β plays an important role in various biological processes such as cell proliferation, apoptosis, differentiation and autophagy, as well as in pathological processes such as inflammation and fibrosis (83). The protective effect of Klotho on inflammation and fibrosis is largely attributed to its ability to block TGF-β.

In the Smad pathway, TGF-β binds to TβRI and TβRII, which form heterodimers, and then induces the phosphorylation of downstream Smad2 and Smad3, forming a complex with Smad4 to enter the nucleus and regulate the transcription of specific genes (84–86). First discovered in 2011, Klotho can directly bind to TβRII and simultaneously inhibit TGF-β1 binding to interfere with TGF-β1 signaling (87). This inhibits the phosphorylation of Smad3, the transactivation of Smad response reporter genes, and the binding of TGF-β1 to the surface of renal and lung epithelial cells in a dose-dependent manner (87). It also alleviates the increased expression of mesenchymal markers and the decreased epithelial marker expression that results from the TGF-β1-induced effect as well as exerting an endogenous anti-EMT effect. Further investigation (88) revealed that TGF-β was suppressed in five distinct cell types including normal rat kidney interstitial fibroblast cells (NRK-49F), human proximal tubular cells (HKC-8), mouse primary renal tubular cells, rat primary cardiomyocytes, and cardiac fibroblasts by the Klotho-derived peptide 1 (KP1). Then in 2022, the study found in C2C12 myotubes, c-a-Klotho binds to type I serine/threonine kinase receptors (ALK5 and ALK4) and type II serine/threonine kinase receptors (ActIIRA and ActRIIB) (89), inhibits various TGF-β signaling activities (myo-growth factor, GDF11, and activin) and also restores the protein levels of differentiation markers (fast MyHC, creatine kinase, and myogenin) and fusion markers (myomaker and myomerer) in C2C12 myotubes (89).

Crucial mechanisms in the regulation of inflammatory responses during an infection encompass epigenetic modifications, which can also be targeted for the modulation of Klotho. Following an inflammatory insult, there is a substantial increase in TGF-β levels, and TGF-β1 exerts influence on miR-34a to enhance its expression in a p53-dependent fashion and target the 3’UTR of Klotho, thereby impacting its transcription (90). KP1 demonstrated the capability to reinstate the expression of long noncoding RNA (lncRNA)-TUG1 and Klotho in fibrotic kidneys, suppress the expression of fibronectin, collagen I, and α-SMA, as well as diminish the levels of p21, p16, and γ-H2AX in fibrotic kidneys through inhibition of the TGF-β/Smad3/miR-223-3p pathway (91). Concurrently, TGF-β induces aberrantly elevated expression of DNA methyltransferase1/3a (DNMT1/3a) via suppression of miR-152 and miR-30, resulting in hypermethylation of the Klotho promoter (92, 93). The utilization of herbal components such as epigallocatechin-3-gallate (EGCG) (92), docosahexaenoic acid (DHA) (93), or DNA methylase inhibitors can effectively impede the abnormally heightened expression of the DNA methylation transferases DNMT1 and DNMT3a, thereby alleviating Klotho promoter hypermethylation and subsequent down-regulation of Klotho. In the realm of histone modification, TGF-β/Smad2/3 signaling induces an aberrant upregulation of HDAC3, leading to the formation of a transcription inhibition complex with nuclear receptor co-inhibitor (NcoR) and NF-κB. This complex not only regulates the acetylation level at H3K4, H3K9, and H4K5 sites but also binds to the Klotho promoter (GAATTCCC: NF-κB binding site), resulting in deacetylation of PPARγ as well as corresponding Klotho promoter histones. Consequently, this leads to a reduction in Klotho expression (94). HDAC8 may directly or indirectly stimulate the up-regulation of TGF-β/Smad3 expression and suppress the expression of BMP-7 and Klotho in injured kidneys (95). Conversely, broad-spectrum HDAC and specific HDAC inhibitors (95–97) have been shown to rejuvenate the anti-renal fibrosis effect of Klotho by facilitating PPARγ acetylation to effectively regulate Klotho, ultimately leading to a significant restoration of Klotho protein levels and successful alleviation of renal and bone injuries associated with renal fibrosis. It has been observed that only inhibition of class I HDAC (HDAC3/8) in the kidney is able to restore Klotho levels, while further investigation is needed to elucidate the detailed mechanism through which HDAC8 interacts with Klotho, as well as whether class II, III, and IV HDACs yield similar effects.

**Figure 3:**
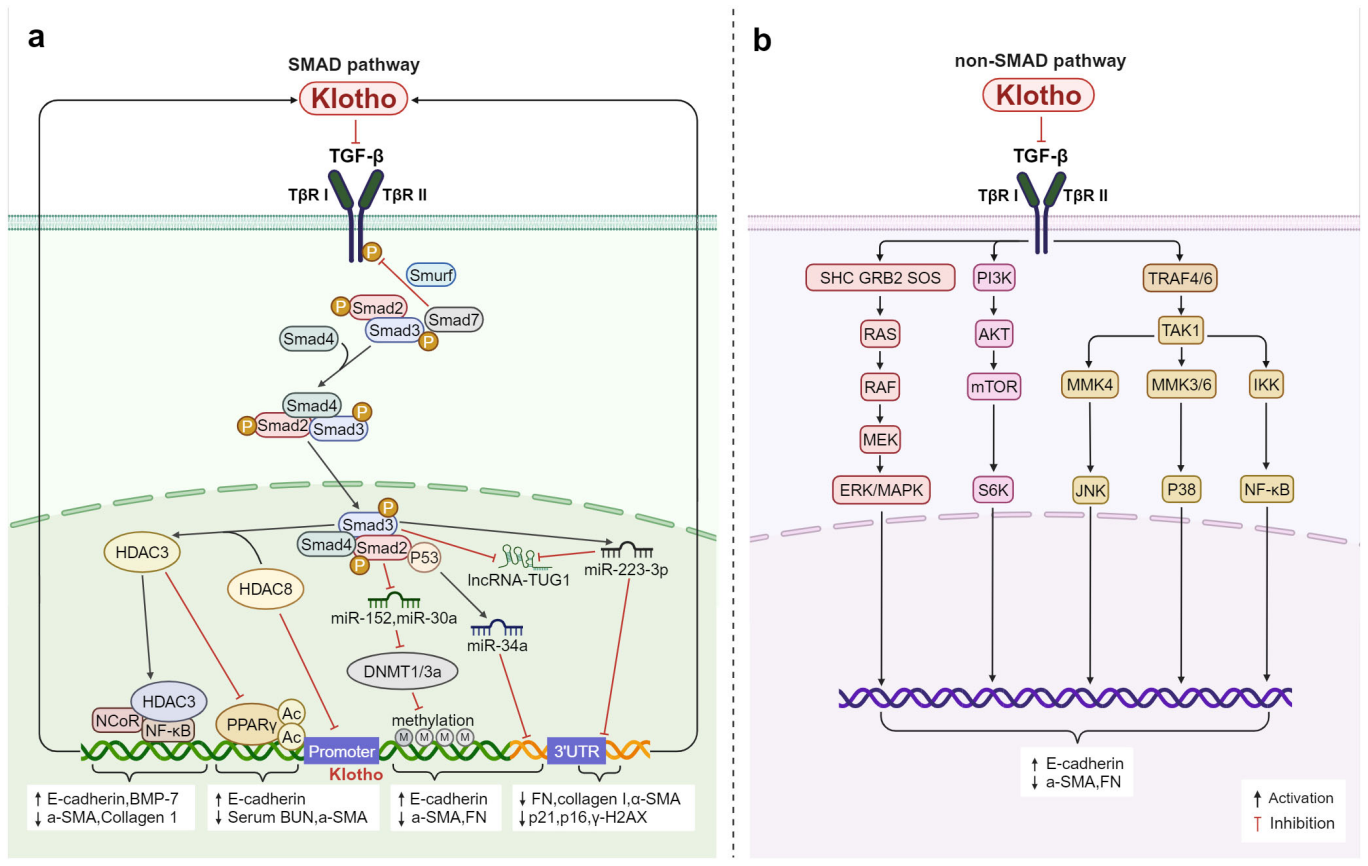
Klotho regulates inflammation and fibrosis through TGF-β: **(A)** In the Smad pathway, Klotho not only inhibits TGF-β activity by binding to TβRI and TβRII, but also interferes with TGF-β expression through regulating methylation, post-translational protein acetylation, and the epigenetic pathways mediated by miRNAs and lncRNAs thereby modulates the inflammation and fibrosis. **(B)** Klotho inhibits TGF-β-induced non-Smad signaling pathways (Ras, MAPK, PI3K, Akt, NF-κB, etc.) and affects the expression of E-cadherin, a-SMA, and FN, regulates inflammation and fibrosis.

Moreover, the TGF-β secreted by inflammatory cells binds to TβR and plays a role in inflammation or fibrosis by transmitting to Ras (98, 99), MAPK (100), phosphatidylinositol 3-kinase (PI3K) (99, 100), serine/threonine protein kinase (AKT) (101), NF-κB (102), etc. Inhibition of the Klotho-dependent TGF-β1/p38MAPK pathway can reduce the levels of a-SMA and FN, increase the expression of E-cadherin, and inhibit the proliferation and fibrosis of abnormal renal cells in diabetic nephropathy (103). More potently than ITD-1, a small molecule inhibitor of TGF-β signaling, KP1 effectively inhibited multiple downstream signals of TGF-β (ERK1/2, JNK, and p38) (88). It also attenuated fibrotic lesions after ischemic or obstructive injury and restored endogenous Klotho expression both *in vivo* and *in vitro*. Therefore, by targeting the TGF-β/Smad non-SMAD signaling pathways, Klotho is capable of functioning as a systemic TGF-β inhibitor that influences fibrosis and inflammation (**Figure 3**).

### 4.3 Inhibition of the TLRs/NF-κB signaling pathway

A family of Toll-like receptors (TLRs), which are an important group of pattern recognition receptors, play a crucial role in the detection of microbial pathogens by human immune cells and in mediating the immune response (104–107). They have the ability to activate the NF-κB pathway, release NF-κB into the nucleus (108, 109), and induce the expression of inflammatory factors such as TNF-α, IL-1β, IL-6, etc., as well as apoptotic factors including Bcl-xL, Bcl2, TRAF1/2, etc. These factors ultimately impact inflammation and fibrosis (110, 111).

Further, TLR4 is the most extensively researched and the first member of the TLR family to be found. After lipopolysaccharide (LPS) binds to TLR4, the signaling pathway is activated, causing MD-2 on the cell surface to form a dimerization complex with TLR4, turning on a series of downstream reactions (112). Klotho targets mature TLR4 and accelerates its degradation through the lysosomal autophagy pathway (111). This further inhibits the phosphorylation of NF-κB p65, lowers the production of ROS, reduces the expression of necrotic apoptotic gene markers, and mitigates AngII-induced necrotic cardiotoxicity. These effects are cardioprotective while also reducing the expression of pro-inflammatory factors (TNF-α and IL-1β) (113). According to The et al. (114), pretreatment with recombinant Klotho reduced the production of MCP-1, IL-6, and ICAM-1 in human aortic valve interstitial cell (AVIC). According to reports, overexpressing Klotho in H_2_O_2_-treated nucleus pulposus (NP) cells significantly decreased the expression of pro-inflammatory cytokines including IL-1β, NOS2, and IL-18 and effectively inhibited TLR4-NF-κB signaling. These results suggest that Klotho attenuates intervertebral disc injury and effectively inhibits H_2_O_2_-induced acute inflammatory responses (115). Extensive research has demonstrated that cytokines, ROS, and DAMPs trigger inflammatory vesicle signaling, elevate IL-1β and IL-18 levels, and result in unbalanced miRNA levels. Additionally, abnormal epigenetic alterations impact the expression of TLRs, NF-κB, and Klotho. In the LPS-induced inflammatory state, miR-199a-5p directly targets the 3′ UTR of Klotho and downregulates its expression. Albumin-stimulated renal tubular epithelial cells induce macrophage M1-type polarization by releasing extracellular vesicles (EVs) containing miR-199a-5p, which targets the Klotho/TLR4 pathway and accelerates diabetic nephropathy progression (116). Another study found that exogenous supplementation of Klotho or inhibition of miR-199a-5p inhibited the activity of the TLR4/NF-κB p65/neutrophil gelatinase-associated lipocalin (NGAL) signaling pathway and reduced the expression of NGAL, fibrosis factors including FN, connective tissue growth factor (CTGF), and inflammatory factors including MCP-1and CXCL5 in response to high glucose stimulation, effectively attenuated the injury of mesangial cells (MCs), and slowed down the progression of diabetic kidney disease (DKD) to end-stage renal disease (117). Therefore, Klotho’s inhibitory effect on the TLRs/NF-κB signaling pathway helps protect tissues from inflammation-induced damage and slows the fibrotic phase of the disease.

**Figure 4:**
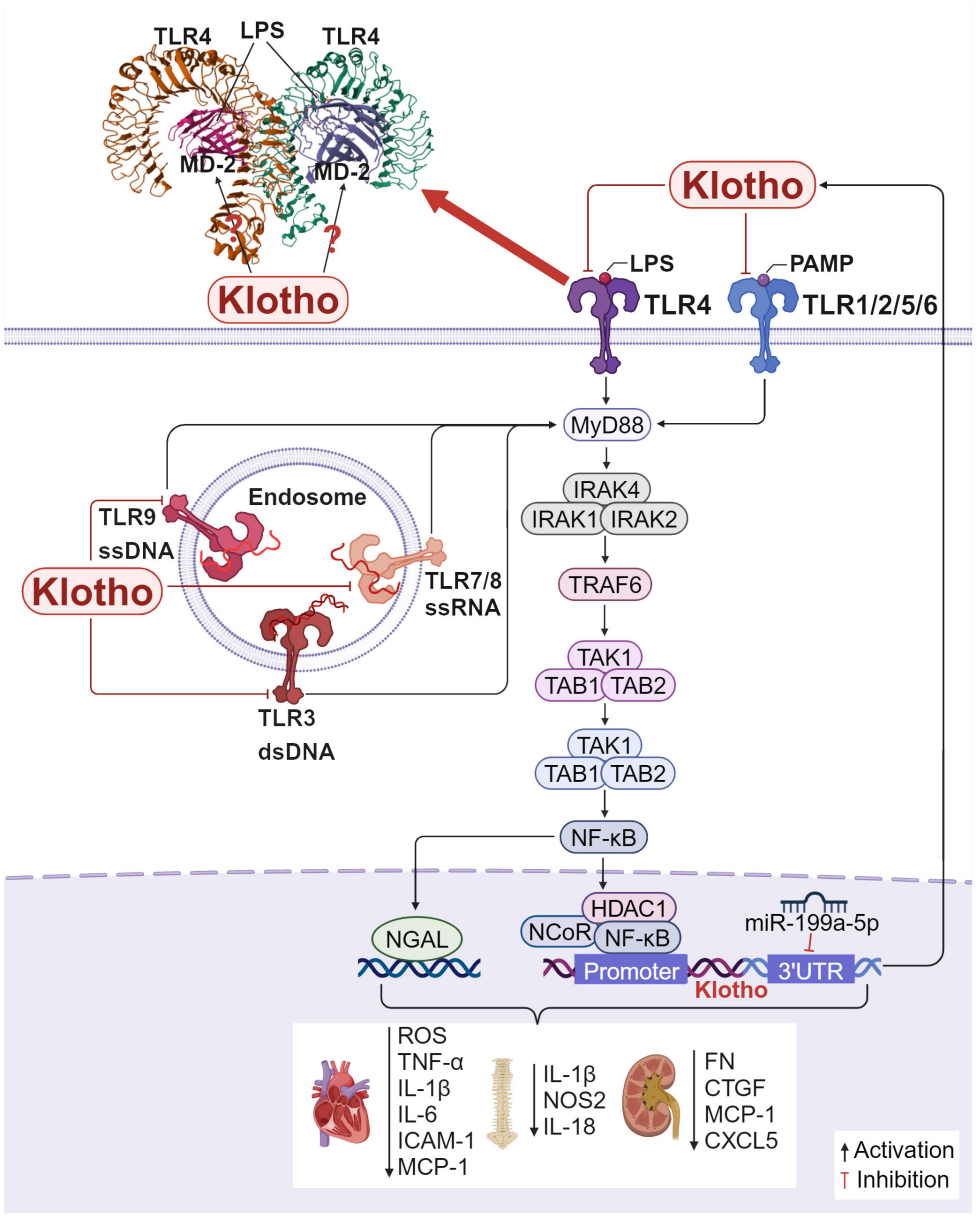
Klotho regulates inflammation and fibrosis through TLRs/NF-κB: Crystal structure of mouse TLR4/MD-2/LPS complex, drawn from PDB accession code 3VQ2 (112). 1) In H9c2 cardiac cells, Klotho inhibits the TLR4/NF-κB pathway, reducing the production of ROS, the expression of necrotic apoptotic gene markers, and the production of MCP-1, IL-6, and ICAM-1, which exerts a protective effect on the heart. 2) In NP cells, overexpressing Klotho inhibits TLR4-NF-κB signaling and decreases the expression of pro-inflammatory cytokines including IL-1β, NOS2, and IL-18, attenuating intervertebral disc injury. 3) In MCs, inhibition of miR-199a-5p or exogenous addition of Klotho inhibits the TLR4/NF-κB p65 signaling pathway, reduces various inflammation and fibrosis related factors.

In conclusion, Klotho’s inhibitory action on the TLRs/NF-κB signaling pathway helps shield tissues from damage caused by inflammation and slows down the disease’s fibrotic phase (**Figure 4**). After NF-κB is activated and translocated into the nucleus, the protein complex containing NF-κB, NCoR, and HDAC1 is recruited to the Klotho promoter at the GAATTCCC (NF-κB binding site) sequence, which inhibits Klotho transcription (118). In situations when there is severe, acute, or chronic inflammation, NF-κB activation may be predominant and Klotho expression is suppressed. Moreover, the TLR4 protein has nine glycosylation sites, and the specific role of Klotho on one or more of these sites remains to be further investigated. In addition to targeting the TLR4 protein and destabilizing it, can Klotho also competitively bind to the TLR4 auxiliary protein MD-2 in the hydrophobic lumen, as known TLR4 inhibitors such as naloxone (119) and naltrexone (120), or the natural product compound curcumin (121), preventing agonists such as LPS from entering the hydrophobic cavity to induce TLR4/MD-2 dimerization, and thus inhibiting the activation of TLR4 and downstream pathways is unknown. Studies addressing whether Klotho can act on TLR4/MD-2 could help develop more effective TLR4 inhibitors to alleviate inflammation and fibrosis. Whether the effects of Klotho on other TLRs are similar to those of TLR4 and whether Klotho can bind to TLRs in the absence of inflammatory injury to participate in other biological processes also need to be further investigated. Therefore, more thorough research is required to determine how to suppress the TLRs/NF-κB pathway through Klotho in order to reduce inflammation and fibrosis.

### 4.4 RAS inhibition of the RAS signaling pathway

Current studies have identified at least five RAS metabolic pathways, which are categorized into classical and nonclassical axes. The classical axis (ACE-Ang-II-AT1R) primarily consists of angiotensin-converting enzyme (ACE), angiotensin worker (Ang-II), and angiotensin II type 1 receptor (AT1R) (122, 123). These axes interact to maintain normal physiological functions. However, various pathological factors can disrupt the balance between these two major axes, leading to hyperactivation of the classical axis. This imbalance can then lead to disruptions in blood pressure regulation and result in oxidative stress, inflammation, and fibrotic damage (124).

**Figure 5:**
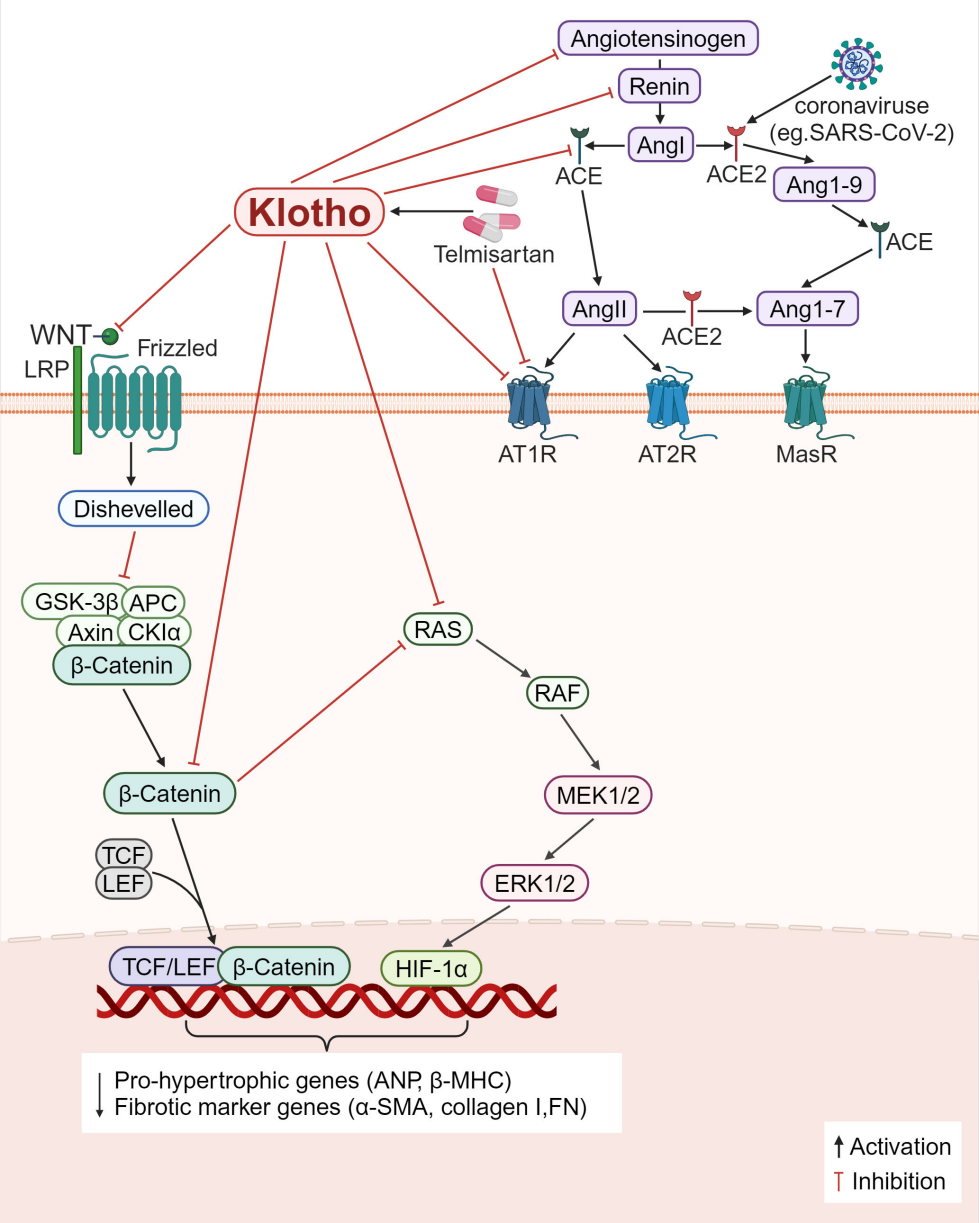
Klotho regulates inflammation and fibrosis through RAS. 1) Klotho directly downregulates the expression of various RAS proteins (angiotensinogen, renin, ACE, AT1R), decreased the levels of Ang II and AGT, decreased the main interstital matrix components (α-SMA, collagen I, FN), reduced the expression of HIF-1α, and alleviated fibrosis. 2) Klotho indirectly blocks RAS proteins by inactivating the Wnt/β-catenin pathway, and modulates inflammation and fibrosis. 3) Some viruses, such as SARS-CoV-2, can bind to ACE2 receptors, and causes ACE2/ACE imbalance, and the AT1R antagonist, Telmisartan, reduces viral replication and ameliorates inflammation and fibrosis.

First, the RAS pathway is directly inhibited by Klotho (**Figure 5**). Klotho reduces renal fibrosis in several chronic kidney disease models, including the 5/6 nephrectomy, unilateral ureteral obstruction (UUO), and adriamycin nephropathy models. It does this by suppressing the expression of several RAS proteins including renin, ACE, AT1R, and angiotensinogen, blocking the levels of TGF-β1 and α-SMA and reducing the number of major interstitial matrix components in the kidney, such as type I collagen and FN (125). β-inhibitory proteins are translocated to the AT1R in response to Ang II activation of the AT1R, which in turn initiates receptor internalization. For the first time, a study by Takenaka et al. (126) demonstrated that Klotho binding to AT1R causes conformational changes in these regions, thereby increasing receptor internalization to decrease the presence of AT1R on human proximal tubules; supplementation of Klotho proteins also lowers blood pressure, kidney Ang II levels, angiotensinogen (AGT) expression, hypoxia induicible factor-1 alpha (HIF-1α) abundance, and levels of medullary fiber connectivity proteins; all these effects attenuate renal medullary fibrosis and improve stress natriuresis. In Ang II-infused mice, Klotho drastically inhibited Ang II-induced cardiomyocyte hypertrophy as well as the proliferation and activation of cardiomyocytes. In the heart, Klotho was found to decrease the ratio of heart weight to tibia length (HW/TL), cardiomyocyte cross-sectional area, fibrotic area, and the expression of pro-hypertrophic genes including atrial natriuretic peptide (ANP) and beta-myosin heavy chain (β-MHC), fibrotic marker genes including α-SMA and collagen-I (127). Second, β-catenin binds to the promoter regions of all RAS component genes and operates on RAS upstream regulators while also modifying the RAS signaling pathway. Furthermore, the promoter regions of all RAS component genes contain binding sites for β-catenin, which acts on upstream regulators of RAS and also modulates the RAS signaling pathway. Klotho can indirectly block RAS genes (AGT, renin, ACE, and AT1), inhibiting inflammation and fibrosis (125) (**Figure 5**).

SARS-CoV-2 is a virus that infects cells through ACE2 as the receptor. Its surface spike protein binds to ACE2 and invades cells, leading to the destruction of ACE2, but has no effect on total ACE production, resulting in ACE/ACE2 imbalance in the body (128). Decreased ACE2 expression impairs the hydrolytic ability of Ang II, resulting in increased Ang II levels and damage to multiple organs through binding to AT1R (129). At the same time, SARS-CoV-2 infected cells release a series of proinflammatory cytokines, leading to a cytokine storm that causes functional failure of multiple organs (130). Duarte et al. (128) found that treatment with telmisartan, a selective AngII receptor (AT1-type) antagonist, could significantly reduce the rate of viral replication and the mortality of patients due to COVID-19. However, whether telmisartan works against other types of viruses, such as SARS-CoV and human coronavirus NL63 (HCoV-NL63), remains to be investigated. Ang II controls the expression of the Klotho gene; chronic renal Ang II treatment in rats results in the downregulation of the Klotho mRNA expression. It is now known that telmisartan is a partial agonist of PPAR-γ, and the Klotho gene is one of the target genes of PPAR-γ action. The application of telmisartan can upregulate the expression of Klotho (131). Further research is required to ascertain if telmisartan or other RAS inhibitors restore SARS-CoV-2-mediated RAS imbalance by upregulating Klotho expression, further (**Figure 5**).

### 4.5 Inhibition of the MAPK signaling pathway

**Figure 6:**
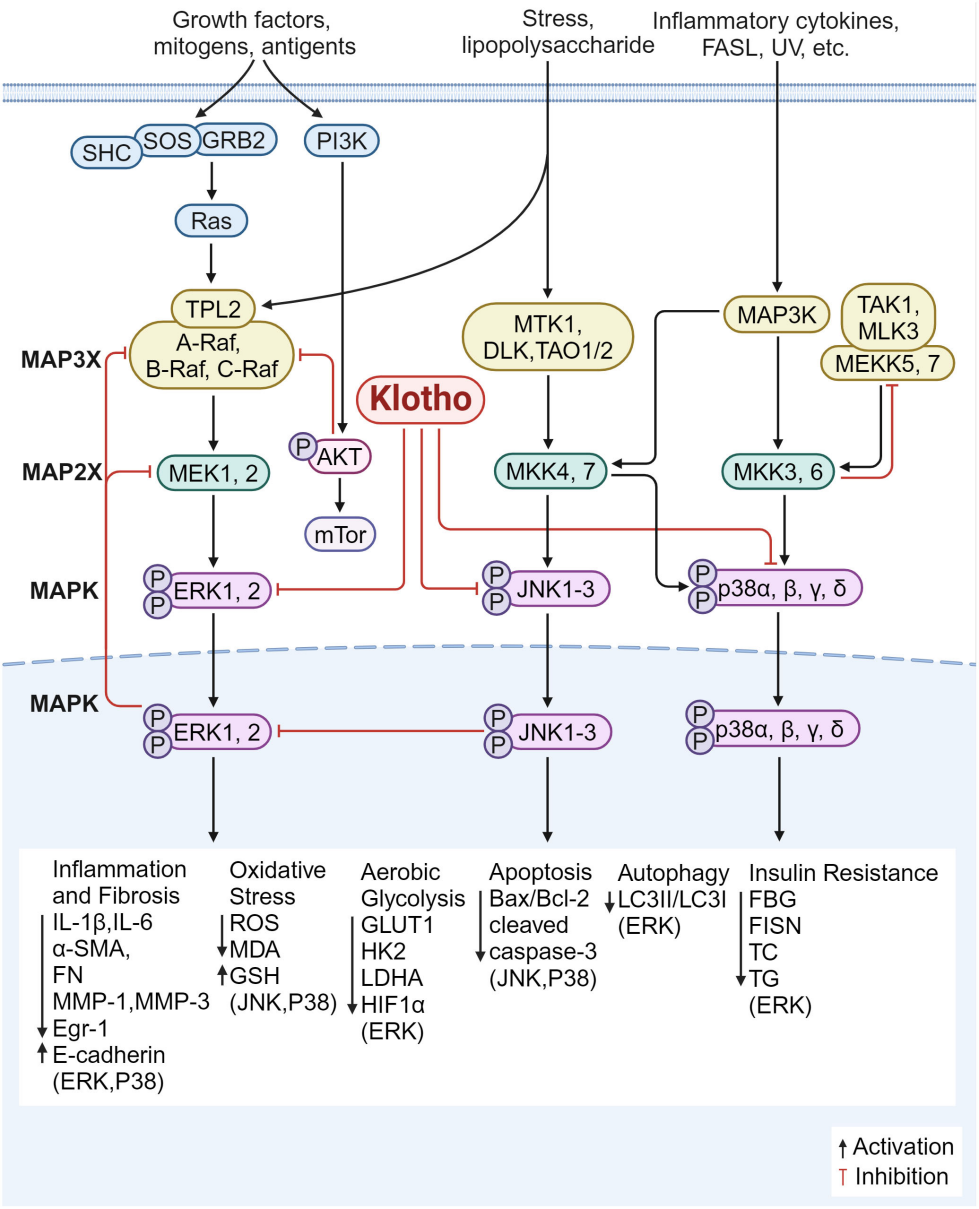
Klotho regulates inflammation and fibrosis through MAPK. 1) The overexpression of Klotho inhibited the ERK pathway, suppressed the expression of glycolytic genes (GLUT1, HK2, LDHA), down-regulated HIF1α level and transcriptional activity; down-regulates the expression of Egr-1 and α-SMA, FN, IL-6, MMP-1 and MMP-3, and up-regulates the expression of E-cadherin; increases the LC3II/LC3I ratio, and increased autophagy; restores the expression of CD29 and p-Akt; improves insulin resistance status; decreased serum levels of FBG, FISN, TC, and TG; inhibited inflammatory oxidative stress injury; and improved fibrosis. 2) Klotho blocks JNK/MAPK and p38/MAPK phosphorylation, decreases cleaved caspase-3 levels and Bax/Bcl-2 ratio, and increases antioxidant enzyme levels and ROS production. 3) Klotho inhibits the P38 pathway, reduces endoplasmic reticulum stress and apoptotic signaling, and downregulates the expression of ANP and BNP; reduces ROS production; decreases the expression of pro-inflammatory factors (IL-1β, IL-6), lowers MDA and increases GSH content; and reduces the expression of α-SMA, E-cadherin, and FN, inhibiting inflammatory responses and improving fibrosis.

Further, the class of serine-threonine protein kinases known as MAPK pathway controls a variety of biological processes, such as gene expression, cell division, proliferation, and apoptosis. ERK, p38 mitogen activated protein kinase (p38MAPK), and JNK are the mammalian MAPK isoforms that have been investigated the most (132). It has been demonstrated that Klotho controls fibrosis and inflammation via these three mechanisms (**Figure 6**).

#### 4.5.1 Inhibition of the ERK1/2 signaling pathway

Klotho overexpression inhibits ERK activation, downregulates transcriptional activity and HIF1α protein levels, decreases the expression of glycolytic genes including glucose transporter type 1 (Glut1), hexokinase 2 (HK2), lactate dehydrogenase A (LDHA), enhances mitochondrial respiration, and improves fibrosis (133). In DKD, Klotho was able to downregulate the expression of Egr-1 and pro-fibrotic genes (α-SMA and FN) and upregulate the expression of E-cadherin. This was achieved by blocking ERK1/2 signaling in HG-treated and TGF-β1-treated HK2 cells (134). In the meantime, overexpression of Klotho can prevent tubular damage caused by hyperglycemia by enhancing autophagy in renal tubular cells, raising the LC3II/LC3I ratio, and inhibiting the AMP-activated protein kinase (AMPK)/ERK pathway (135). According to Li JM et al. (136), overexpressing α-Klotho in cardiac fibroblasts improved diabetic cardiomyopathy fibrosis, attenuated cardiac mesenchymal and perivascular collagen deposition, partially reversed ERK1/2 activation, and ameliorated the state of insulin resistance. It also reduced the serum levels of FBG, FISN, TC, and TG. Furthermore, Klotho supplementation reduces the expression of IL-6 and ROS in fibroblasts from patients with pelvic floor prolapse (POP); by reducing ERK1/2 phosphorylation, Klotho can also downregulate the expression of MMP-1 and MMP-3, thereby increasing the resistance of fibroblasts to oxidative stress and suppressing inflammatory responses (137). These findings imply that Klotho modulates the ERK1/2 signaling pathway, which reduces inflammation and fibrosis via regulating insulin resistance, glycolysis, inflammatory oxidative stress injury, and autophagy.

#### 4.5.2 Inhibition of the JNK signaling pathway

The JNK signaling pathway can be activated by various stimuli, such as inflammatory factors and oxidative stress, translocation from cytoplasm to nucleus activates c-Jun (138) to form homologous or heterodimer with activated transcription factor (ATF) protein to regulate target protein transcription (139). Activated JNK also increases the release of pro-apoptotic factors in mitochondria, leading to apoptosis.

Increased glycolysis resulted from JNK being overactivated in spermatogonial stem cells (SSCs) knocked down for Klotho. According to Park SJ et al. (140), Klotho mutant mice showed marked memory deficits when compared to wild-type mice; in the hippocampal regions, the mutation exacerbated neuroinflammation by downregulating the expression of cell death/pro-apoptotic factors including p-JNK, Bcl-2-associated X protein (BAX), cleaved cyst caspase-3 and increasing the expression of cell survival/anti-apoptotic factors including phosphorylated AKT (p-AKT)/phosphorylated-glycogen synthase kinase 3 beta (p-GSK3-β), p-ERK, and B-cell lymphoma-2 (Bcl-2). Additionally, it was reported that exogenous Klotho protein or overexpression of Klotho blocked phosphorylation of JNK/MAPK and p38/MAPK in mouse kidney tubular epithelium cell line (TCMK-1) cells, reduced the amount of cleaved caspase-3 and the ratio of Bax/Bcl-2, increased the production of ROS and antioxidant enzymes, controlled the mitochondrial function of renal tubular epithelial cells, and attenuated renal injury (141).

#### 4.5.3 Inhibition of the P38 signaling pathway

p38α (MAPK14), p38β (MAPK11), p38γ (MAPK12), and p38δ (MAPK13) are the four members of the p38MAPK family. When oxidative stress is activated, reactive ROS are produced. These ROS activate p38 MAPK, attract inflammatory cells, and release pro-inflammatory proteins, which exacerbate fibrosis and cause an inflammatory response. By controlling the apoptosis signal-regulating kinase 1 (ASK1)/p38 MAPK signaling pathway, Klotho has been shown to reduce oxidative stress and shield dopaminergic neurons from oxidative damage (77, 142). In cerebrally infarcted rats, Klotho has also been shown to improve neurological function, reduce ischemic injury, and reduce the area of cerebral infarction by inhibiting the p38 MAPK pathway by downregulating the expression of aquaporin 4 (AQP4) (143). The involvement of p38-δ in neurodegenerative and inflammatory illnesses has been identified recently; however, it is not yet known if Klotho regulates by inhibiting p38-δ. Klotho suppression of the p38 pathway decreases ROS produced by cardiomyocytes, improving cardiac disease and reducing myocardial fibrosis; it also decreases endoplasmic reticulum stress and apoptotic signaling, and downregulates the production of ANP and B-type natriuretic peptide (BNP) (144). Zhang et al. (145) found that by blocking ROS/P38 MAPK signaling, Klotho could inhibit the expression of pro-inflammatory factors (IL-1β, IL-6), reduce ROS, lower MDA, and increase glutathione (GSH) content. Klotho treatment also reduced mitochondria-dependent apoptosis of lung epithelial cells and attenuated lung tissue injury. These findings imply that Klotho’s antioxidant and anti-apoptotic properties prevent p38MAPK signaling, lower ROS production, and reduce inflammation and fibrosis in the nerves and organs such as the kidneys, heart, and lungs.

## 5 Strategies for enhancing Klotho levels

**Figure 7:**
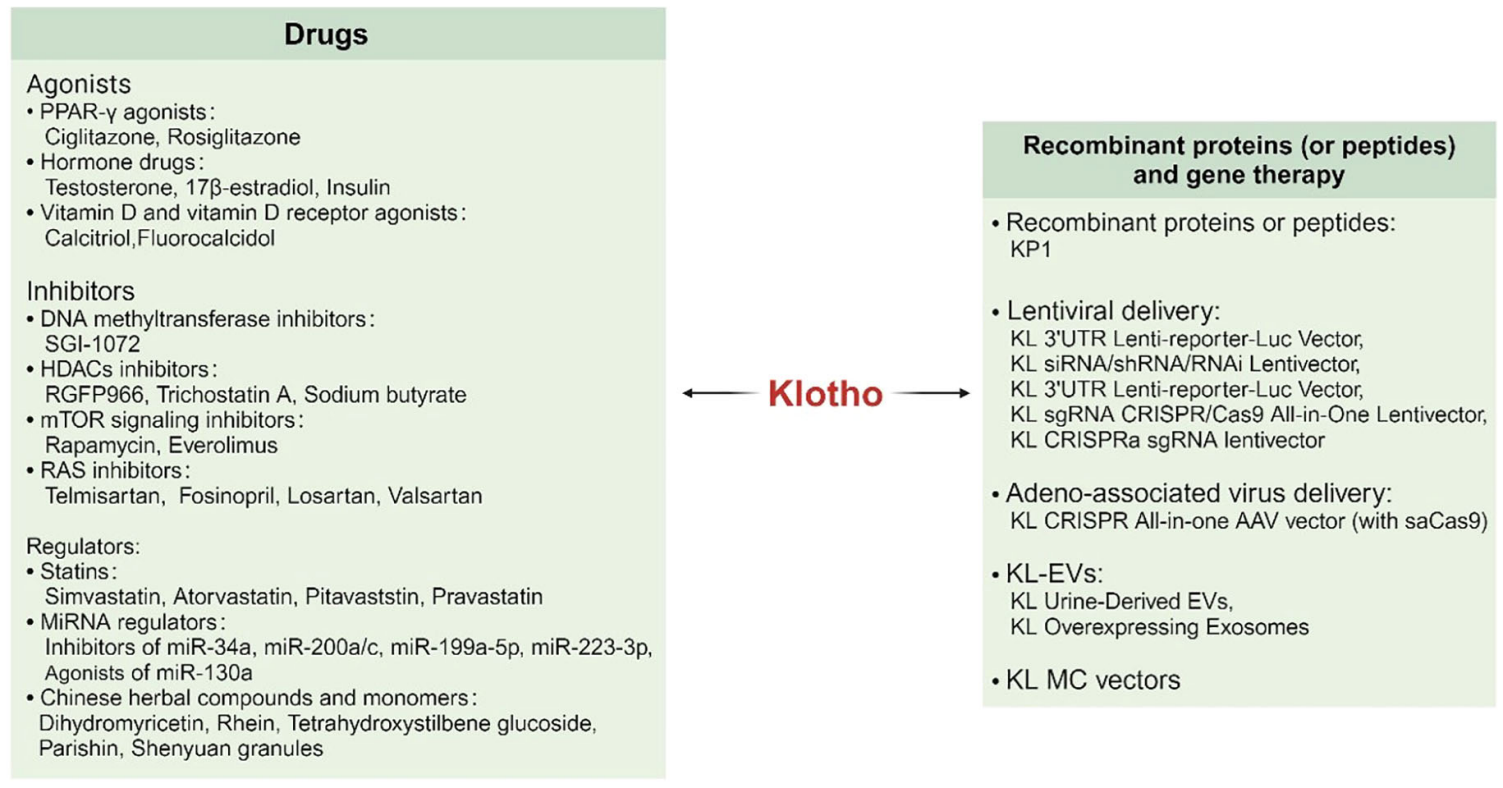
Drugs, recombinant proteins (or peptides) and gene therapy targeting Klotho.

Various approaches have been explored to increase Klotho levels, including the use of drugs, recombinant proteins (or peptides), and gene therapies in preclinical models of the disease (**Figure 7**). These intriguing discoveries are summarized in this section.

### 5.1 Drugs that enhancing Klotho

Drugs that have been found to increase Klotho levels include agonists and inhibitors. For example, PPAR-γ upregulates Klotho through its atypical response in the 5′ flanking region of the Klotho gene (146). Additionally, androgens upregulate Klotho by increasing nuclear androgen receptor (AR) expression, which then upregulates Klotho through androgen response elements (AREs) (147). Klotho expression is likewise increased by estrogens. Both *in vivo* and *in vitro*, estrogen has been shown to alter Kl expression in rat hippocampus neurons. Since the hippocampus is a key component in the regulation of the stress system, lowering the amounts of Klotho protein in the rat hippocampal tissue led to a reduction in stress recovery, with this effect being more noticeable in female rats (148). In addition to animals, estrogens in plants also induce Klotho transcriptional activation, e.g., genistein )a phytoestrogenic isoflavone enriched in dietary soy products(protects Klotho levels and mitigates renal fibrosis in UUO mice by reversing HDAC3 deacetylation of the Klotho promoter while inhibiting aberrant expression of DNMT1/3a (149). While insulin promotes Klotho production possibly by inducing protein hydrolytic activity of ADLs and ADAM10/17 (27). The use of prohormonal drugs may indirectly enhance Klotho expression. Upon activation of vitamin D, the vitamin D receptor (VDR) forms a heterodimer with the retinol X receptor (RXR) and translocates to the nucleus. In the nucleus, it binds to the vitamin D response element (VDRE) within the promoter region of the Klotho gene and induces its expression (150). Another class of drugs that enhances Klotho expression is the use of vitamin D receptor agonists.

Inhibitors of DNA methyltransferase demonstrate the capacity to upregulate Klotho by counteracting the hypermethylation-induced downregulation of the Klotho promoter (92, 93); HDCA broad-spectrum and specific inhibitors effectively intervene to restore Klotho levels through protein acetylation (94, 103, 151). Furthermore, inhibitors targeting various inflammatory pathways, such as mTOR signaling inhibitors, have been shown to upregulate Klotho expression by inhibiting the Toll signaling pathway (152–154), while RAS inhibitors have been found to elevate Klotho levels by mitigating the inhibitory effect of Ang-II on Klotho expression (130, 155, 156).

Statins have been discovered to augment the expression of Klotho mRNA through the inhibition of Rho/Rho-kinase and activation of the FOXO pathway (157–160). The regulation of Klotho expression by miRNAs is multifaceted. MiR-34a (90), miR-199a-5p (117), and miR-223-3p (91) all bind to the 3’-UTR of Klotho mRNA, resulting in its downregulation, whereas miR-130a exerts the opposite effect (161). Hence, a variety of miRNAs can be utilized to modulate the level of Klotho; moreover, certain Chinese herbal compounds and monomers have demonstrated efficacy in elevating Klotho expression and mitigating inflammation and fibrosis (90, 111, 162–164).

### 5.2 Enhancement of Klotho through recombinant proteins and gene therapy

In addition to pharmaceutical induction, the supplementation of recombinant Klotho proteins (or peptides) directly addresses protein deficiencies within the body. Studies have shown that the introduction of recombinant Klotho (rKL) protein at a cellular level can mitigate inflammation in various organs such as the kidneys (165, 166), heart (167), bone (168), eyes (169), and more. Furthermore, KP1 has displayed promising potential by alleviating SARS-CoV-2 N protein-induced HK-2 cell senescence and apoptosis, reducing markers of epithelial-mesenchymal transition, and attenuating renal tubular injury (170). However, due to its relatively large size and distribution of positive and negative charges on its surface, direct administration of naked Klotho protein may face challenges in penetrating cell membranes efficiently. This limitation could impact its clinical application. Henceforth, commencing with gene therapy utilizing lentivirus and adenovirus as vector delivery systems for the promotion of the Klotho gene, siRNA, shRNA, RNAi, and CRISPR-Cas9 constructs present more viable options for Klotho treatment. Nevertheless, in light of potential toxicity risks, insertional mutagenesis, and off-target effects associated with viral vectors, there is a pressing need to explore natural endogenous non-viral vectors for the transport of Klotho that offer increased biocompatibility, reduced immunogenicity, greater capacity, enhanced stability and efficiency in transportation while also being cost-effective. Extracellular vesicles (EVs) serve as intrinsic carriers for intercellular transfer of biological information and possess the distinct advantage of traversing the blood-brain barrier. This has emerged as a novel cell-free therapeutic strategy. The utilization of urine-derived EVs (uEVs) for transporting recombinant Klotho proteins (171) and exosomal transportation of sKL by MSCs (172) have both demonstrated significant restoration in endogenous Klotho expression and heightened accumulation within target tissues. The unique transport mechanism offered by Klotho-EVs holds promise in improving hormonal drug therapy for fibrotic diseases that may otherwise result in adverse clinical outcomes. Minicircle (MC), a category of non-viral DNA vectors carrying cassette sequences encoding Klotho genes successfully achieved self-production of Klotho protein in HEK293T cells. The therapeutic efficacy of Klotho-MC was validated in ischemia-reperfusion (I/R) injury and UUO (173). Further exploration into emerging carriers such as contractile injection systems (CISs) utilizing commensal bacteria (174), non-viral nanocarriers including lipid nanoparticles (LNPs) (175), polymer nanoparticles (176), alongside other innovative carriers for future research on transporting Klotho will lend support to its integration into diagnostic and therapeutic approaches pertaining to inflammatory and fibrotic diseases.

## 6 Discussions and future prospects

Since the anti-aging protein Klotho was first reported in 1997, much progress has been made regarding the role of Klotho protein in pathological and physiological conditions. Klotho in humans can be divided into α-Klotho, β-Klotho, and γ-Klotho according to its structure. At present, most studies are focused on the first two subtypes, and the role and mechanism of γ-Klotho are poorly understood. α-Klotho and β-Klotho exist as co-receptors of FGFRs or secretory forms and are involved in the pathophysiology of a variety of human diseases, including genetic diseases, dysplastic diseases, metabolic disorders, degenerative diseases, and injury and regeneration. In its pathological state, Klotho regulates inflammation and fibrosis by controlling immune cells and cytokines in a variety of tissues and organs via distinct signaling pathways. In this review, we reviewed the structure, role, and regulatory mechanism of Klotho in inflammation and fibrosis.

The roles of Klotho are related to various factors, and its function is strictly controlled, with different transduction specificity, which mainly depends on the molecular structure and existence mode of Klotho. Membrane klotho, also known as FGFR co-receptor, forms a complex with FGFR and transmits various signals to the cell while binding to FGF. Interestingly, the FGF bound by different subtypes of Klotho is different from the FGER subtype, which may be related to its molecular structure and key amino acids at the binding site. Interestingly, different subtypes of klotho bind to different subtypes of FGER or FGF, which may be related to their molecular structure and key amino acids at the binding site. This difference can lead to completely different signaling pathways. The secreted form of Klotho acts similarly to hormones and can affect surrounding cells and tissues. A few recent studies have demonstrated that TGFR can bind to Klotho, and the importance of the TGF-β/TGFR pathway in inflammatory response and fibrosis is well elucidated. In addition, Klotho can regulate various important signaling pathways, such as TLR/NF-κB, RAS, and MAPK, and ameliorate tissue fibrosis. In the immune system, Klotho can regulate the polarization of macrophages by regulating TLR and TGF-β signaling pathways, and alleviate the onset of inflammation. Moreover, Klotho can also affect the development of lymphocytes. Even though there are still many mechanisms of Klotho to be explored, especially the expression pattern and precise role of Klotho in the inflammatory response and fibrosis during the process of different diseases/injuries. However, except for FGFR and TGFR, little is known about the specific receptor for Klotho. The current impediment to further exploration lies in the elucidation of how Klotho interacts with target cells in a pathological state, as well as the assessment of Klotho’s binding affinity to various molecules. Additionally, the utilization of ELISA for Immuno-Biological Laboratories (IBL) in clinical databases such as Nutrition Examination Surveys (NHANES), has led to a substantial number of studies opting for time-resolved fluorescence immunoassay (TRF) for their assays. Discrepancies in epitopes recognizing soluble Klotho across different assays have resulted in significant variations in measured sKL levels. Moreover, measurements of Klotho levels in blood, cerebrospinal fluid, and urine exhibit variability among different diseases. Moving forward, it is imperative to establish a standardized method for quantifying Klotho and devise a grading scale corresponding to each disease type to ascertain the patient’s disease status. Furthermore, there is an urgent need for a comprehensive comparison regarding the indications, efficacy, safety, and individual variability associated with diverse drug therapies aimed at enhancing Klotho levels. Additionally, efforts should be directed towards exploring more robust methods for delivering Klotho *in vivo* to facilitate its transition from preclinical research to clinical applications. Finally, currently available experimental data on Klotho are from small observational studies, and data on Klotho are missing or missing from some clinical databases. Future clinical validation will require more comprehensive prospective large-sample multicenter studies, and patients should be routinely followed up to obtain biological samples and clinical data. Such studies will likely support future advancements in the treatment of fibrotic diseases and the development of appealing and novel therapeutic approaches.
